# Bis[*N*-(4-chloro­phen­yl)pyridine-3-carboxamide]­silver(I) nitrate

**DOI:** 10.1107/S1600536810025511

**Published:** 2010-07-03

**Authors:** Chun-Yue Shi, Chun-Hua Ge, Qi-Tao Liu

**Affiliations:** a Department of Chemistry, Northeast Normal University, Changchun 130024, People’s Republic of China; b Applied Chemistry Department, Shenyang University of Chemical Technology, Shenyang 110142, People’s Republic of China; c College of Chemistry, Liaoning University, Shenyang 110036, People’s Republic of China

## Abstract

In the title compound, [Ag(C_12_H_9_ClN_2_O)_2_]NO_3_, two N atoms from two pyridine rings of two *N*-(4-chloro­phen­yl)pyridine-3-carboxamide ligands coordinate to the Ag^I^ atom, forming a nearly linear geometry with an N—Ag—N angle of 173.41 (7)°. The crystal structure is stabilized by N—H⋯O, C—H⋯O and C—H⋯Cl hydrogen bonds and π–π stacking inter­actions [centroid–centroid distance = 3.5469 (16) Å] between the pyridyl and benzene rings. The shortest Ag⋯Ag distance is 3.2574 (5) Å.

## Related literature

For general background to metal-organic complexes with pyridyl carboxamide ligands, see: Noveron *et al.* (2002 ▶); Zhang *et al.* (2002 ▶); Mondal *et al.* (2004 ▶); Jacob & Mukherjee (2006 ▶). For related structures and the synthesis of the title ligand, see: Shi *et al.* (2007 ▶, 2008 ▶).
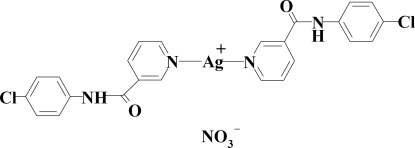


## Experimental

### 

#### Crystal data

[Ag(C_12_H_9_ClN_2_O)_2_]NO_3_
*M* *_r_* = 635.20
Triclinic, 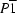

*a* = 10.0745 (10) Å
*b* = 10.1425 (10) Å
*c* = 13.473 (2) Å
α = 107.515 (2)°
β = 102.602 (2)°
γ = 103.706 (1)°
*V* = 1211.6 (2) Å^3^
*Z* = 2
Mo *K*α radiation
μ = 1.10 mm^−1^
*T* = 296 K
0.24 × 0.23 × 0.18 mm

#### Data collection

Bruker APEXII CCD area-detector diffractometer
Absorption correction: multi-scan (*SADABS*; Sheldrick, 1996 ▶) *T*_min_ = 0.776, *T*_max_ = 0.820
6194 measured reflections
4232 independent reflections
3848 reflections with *I* > 2σ(*I*)
*R*_int_ = 0.014

#### Refinement

*R*[*F* ^2^ > 2σ(*F* ^2^)] = 0.026
*wR*(*F* ^2^) = 0.064
*S* = 1.05
4232 reflections
334 parameters
H-atom parameters constrained
Δρ_max_ = 0.27 e Å^−3^
Δρ_min_ = −0.39 e Å^−3^

### 

Data collection: *APEX2* (Bruker, 2007 ▶); cell refinement: *SAINT* (Bruker, 2007 ▶); data reduction: *SAINT*; program(s) used to solve structure: *SHELXS97* (Sheldrick, 2008 ▶); program(s) used to refine structure: *SHELXL97* (Sheldrick, 2008 ▶); molecular graphics: *SHELXTL* (Sheldrick, 2008 ▶); software used to prepare material for publication: *SHELXL97*.

## Supplementary Material

Crystal structure: contains datablocks I, global. DOI: 10.1107/S1600536810025511/zl2286sup1.cif

Structure factors: contains datablocks I. DOI: 10.1107/S1600536810025511/zl2286Isup2.hkl

Additional supplementary materials:  crystallographic information; 3D view; checkCIF report

## Figures and Tables

**Table 1:** Hydrogen-bond geometry (Å, °)

*D*—H⋯*A*	*D*—H	H⋯*A*	*D*⋯*A*	*D*—H⋯*A*
N2—H1⋯O4	0.86	2.10	2.953 (3)	169
N4—H2⋯O1^i^	0.86	2.10	2.931 (3)	162
C2—H3⋯O5	0.93	2.51	3.210 (3)	133
C3—H4⋯O3^ii^	0.93	2.57	3.300 (3)	136
C4—H5⋯Cl2^iii^	0.93	2.83	3.516 (3)	132
C5—H6⋯O1^iv^	0.93	2.55	3.376 (3)	148
C8—H7⋯O1	0.93	2.27	2.841 (3)	119
C11—H9⋯O2^v^	0.93	2.49	3.194 (4)	132
C16—H13⋯O5^vi^	0.93	2.48	3.370 (4)	160
C20—H15⋯O2	0.93	2.46	2.906 (3)	109

Symmetry codes: (i) 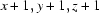; (ii) 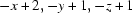; (iii) 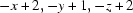; (iv) 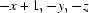; (v) 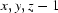; (vi) 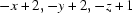.

**Fig. 1.**
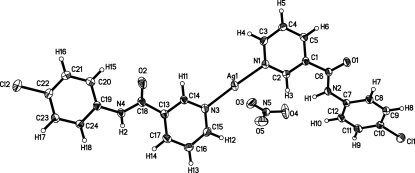
The molecular structure of the title complex with the atom numbering scheme. Displacement ellipsoids are drawn at the 30% probability level.

**Fig. 2.**
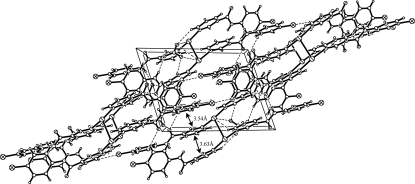
The packing diagram of molecules, viewed down the b axis, with the weak interactions shown as dashed lines and π-π interactions as double arrows.

## supplementary crystallographic information

### Comment

Supramolecular chemistry has generated considerable interest due to the novel structural topologies that can be built that way and due to its potential applications in many areas of science. The carboxamide functionality is an appropriate intermolecular connector, in part due to its well known ability to act as a hydrogen-bonding donor (via the amide hydrogen atoms) or acceptor (via the amide carbonyl oxygen atoms) to enhance structure diversities. Therefore, pyridyl-type compounds that contain a carboxamide group have been used to produce a great number of novel metal-organic complexes (see, for example, Noveron *et al.*, 2002; Zhang *et al.*, 2002; Mondal *et al.*, 2004; Jacob & Mukherjee, 2006). Recently, we have used the non-chelating ligand 3-pyridinecarboxamide in the syntheses of several metal complexes with different topologies (Shi *et al.*, 2007; Shi *et al.*, 2008). In this paper, the crystal structure of the title silver(I) complex is reported.

In the title complex (Fig. 1), each asymmetric unit contains one NO_3_^–^ anion and one [Ag(*N*-(4'-chlorophenyl)-3-pyridinecarboxamide)_2_]^+^ cation. The Ag^I^ ion is coordinated by two nitrogen atoms from two pyridyl rings of two crystallographically independent ligands, thus forming a slightly distorted linear coordination geometry around the silver center. Adjacent symmetry related Ag atoms are connected through nitrate anions via weak interactions with two of the nitrate oxygen atoms (O3 and O5) to form dinuclear units. The distances of Ag···O3^ii^ and Ag···O5 are 2.773 (3) and 2.835 (2) Å, respectively (symmetry operator ii = 2-x,1-y,1-z). The dinculear units are inversion symmetric and the two symmetry related silver ions are bridged in a chelating fashion by two symmetry equivalent nitrate ions. The Ag1···Ag1^ii^ seperation within the units is 3.2574 (5) Å. Via the third oxygen atom the bridging nitrate anion is also hydrogen bonded to one of the amide N—H groups (Table 1). The dimeric units are further stabilized by π-π interactions between pyridyl rings within the dimers [Cg1···Cg2^ii^ = 3.631 (1) Å with a slippage of 1.371 Å, where Cg1 and Cg2 are the centroids of the N1/C1–C5 and N3/C13–C17 pyridyl rings].

The amide unit on the other ligand molecule undergoes a hydrogen bond with one of the amide keto groups in neighboring molecules, which link the dinuclear units together to form infinite 1-D chains via double N—H···O hydrogen bonds [N4···O1^i^ = 2.931 (3) Å, symmetry operator i: x+1, y+1, z+1, Table 1].

The infinite parallel hydrogen bonded chains of complexes are further connected through non-classical hydrogen bonds (Table 1) to generate a 2-D sheet-like network (Fig. 2). These sheets are ultimately joined together to form a 3-D solid network by additional hydrogen bonds and π-π stacking interactions between the pyridyl and benzene rings of neighboring ligands [Cg2···Cg4^v^ (Symmetry operator v: -x+2, -y+2, -z+2) = 3.5469 (16) Å with a slippage of 0.082 Å, where Cg2 and Cg4 are the centroids of the N3/C13–C17 pyridyl and C19–C24 benzene rings].

### Experimental

*N*-(4'-chlorophenyl)-3-pyridinecarboxamide was prepared from nicotinoyl chloride hydrochloride and 4-chloroaniline in the presence of triethylamine, yield 80% (Shi *et al.*, 2008). An ethanolic solution of the organic ligand (0.5 mmol in 20 ml ethanol) was added dropwise to AgNO_3_ (0.5 mmol in 5 ml water). The resulting mixture was stirred for 20 min at room temperature and was then filtered. Single crystals suitable for data collection were obtained by slow evaporation of the solvent in a dark room (0.12g, yield 67%). *M*.P.: 345-346K. ^1^H NMR (d_6_-DMSO ): δ 10.48 (s, 1H, H1), 9.09 (s, 1H, H3), 8.73 (d, 1H, H4), 8.28 (d, 1H, H6), 7.80 (d, 2H, H7, H10), 7.54 (m, 1H, H5), 7.36 (d, 2H, H8, H9). IR (KBr)/cm^-1^: 701, 724, 833, 1093, 1329, 1351, 1398, 1489, 1535, 1604, 1650, 1680, 3067, 3276.

### Refinement

The H atoms bound to the N atoms were located in a difference Fourier map and refined with a distance restraint of 0.87 (2) Å. All other H atoms were positioned geometrically and refined using a riding model, with C—H = 0.93–0.97 Å, O—H = 0.84 Å and with *U*_iso_(H) = 1.2 *U*_eq_(C) or 1.5 *U*_eq_(C, O) for methyl and hydroxy groups.

### Crystal data

**Table:**

[Ag(C_12_H_9_ClN_2_O)_2_]NO_3_	*Z* = 2
*M**_r_* = 635.20	*F*(000) = 636
Triclinic, *P*1	*D*_x_ = 1.741 Mg m^−^^3^
Hall symbol: -P 1	Mo *K*α radiation, λ = 0.71073 Å
*a* = 10.0745 (10) Å	Cell parameters from 4853 reflections
*b* = 10.1425 (10) Å	θ = 2.2–27.8°
*c* = 13.473 (2) Å	µ = 1.10 mm^−^^1^
α = 107.515 (2)°	*T* = 296 K
β = 102.602 (2)°	Block, colourless
γ = 103.706 (1)°	0.24 × 0.23 × 0.18 mm
*V* = 1211.6 (2) Å^3^	

### Data collection

**Table:**

Bruker APEXII CCD area-detector diffractometer	4232 independent reflections
Radiation source: fine-focus sealed tube	3848 reflections with *I* > 2σ(*I*)
graphite	*R*_int_ = 0.014
phi and ω scans	θ_max_ = 25.0°, θ_min_ = 2.2°
Absorption correction: multi-scan (*SADABS*; Sheldrick, 1996)	*h* = −11→11
*T*_min_ = 0.776, *T*_max_ = 0.820	*k* = −12→11
6194 measured reflections	*l* = −7→16

### Refinement

**Table:**

Refinement on *F*^2^	Primary atom site location: structure-invariant direct methods
Least-squares matrix: full	Secondary atom site location: difference Fourier map
*R*[*F*^2^ > 2σ(*F*^2^)] = 0.026	Hydrogen site location: inferred from neighbouring sites
*wR*(*F*^2^) = 0.064	H-atom parameters constrained
*S* = 1.05	*w* = 1/[σ^2^(*F*_o_^2^) + (0.0278*P*)^2^ + 0.6379*P*] where *P* = (*F*_o_^2^ + 2*F*_c_^2^)/3
4232 reflections	(Δ/σ)_max_ = 0.002
334 parameters	Δρ_max_ = 0.27 e Å^−^^3^
0 restraints	Δρ_min_ = −0.39 e Å^−^^3^

### Special details

**Table:**

Geometry. All e.s.d.'s (except the e.s.d. in the dihedral angle between two l.s. planes) are estimated using the full covariance matrix. The cell e.s.d.'s are taken into account individually in the estimation of e.s.d.'s in distances, angles and torsion angles; correlations between e.s.d.'s in cell parameters are only used when they are defined by crystal symmetry. An approximate (isotropic) treatment of cell e.s.d.'s is used for estimating e.s.d.'s involving l.s. planes.
Refinement. Refinement of *F*^2^ against ALL reflections. The weighted *R*-factor *wR* and goodness of fit *S* are based on *F*^2^, conventional *R*-factors *R* are based on *F*, with *F* set to zero for negative *F*^2^. The threshold expression of *F*^2^ > σ(*F*^2^) is used only for calculating *R*-factors(gt) *etc*. and is not relevant to the choice of reflections for refinement. *R*-factors based on *F*^2^ are statistically about twice as large as those based on *F*, and *R*- factors based on ALL data will be even larger.

### Fractional atomic coordinates and isotropic or equivalent isotropic displacement parameters (Å^2^)

**Table:**

	*x*	*y*	*z*	*U*_iso_*/*U*_eq_	
Ag1	0.87169 (2)	0.56734 (2)	0.518117 (14)	0.04869 (8)	
Cl1	0.58438 (8)	0.45918 (8)	−0.36615 (5)	0.05917 (18)	
Cl2	1.33119 (13)	1.05319 (10)	1.51069 (6)	0.0891 (3)	
N2	0.64188 (19)	0.3727 (2)	0.05514 (14)	0.0375 (4)	
H1	0.7178	0.4325	0.1077	0.045*	
O1	0.44206 (18)	0.17952 (18)	0.01256 (13)	0.0507 (4)	
N3	0.9912 (2)	0.7669 (2)	0.65614 (15)	0.0402 (4)	
N1	0.7302 (2)	0.3744 (2)	0.38083 (14)	0.0388 (4)	
C6	0.5572 (2)	0.2683 (2)	0.07732 (17)	0.0356 (5)	
C7	0.6197 (2)	0.3948 (2)	−0.04546 (17)	0.0347 (5)	
N4	1.2066 (2)	0.9810 (2)	1.04496 (15)	0.0417 (5)	
H2	1.2615	1.0478	1.0314	0.050*	
C8	0.4900 (2)	0.3301 (3)	−0.12996 (18)	0.0406 (5)	
H7	0.4103	0.2727	−0.1208	0.049*	
C3	0.7042 (2)	0.2420 (3)	0.38709 (19)	0.0422 (5)	
H4	0.7352	0.2354	0.4551	0.051*	
C2	0.6833 (2)	0.3825 (2)	0.28185 (17)	0.0355 (5)	
H3	0.6993	0.4742	0.2771	0.043*	
C13	1.0816 (2)	0.8926 (2)	0.85119 (18)	0.0355 (5)	
O2	1.0207 (2)	0.7703 (2)	0.96688 (15)	0.0770 (7)	
C16	1.0810 (3)	1.0276 (3)	0.7337 (2)	0.0457 (6)	
H13	1.1003	1.1159	0.7233	0.055*	
C5	0.5870 (2)	0.1236 (2)	0.19497 (18)	0.0378 (5)	
H6	0.5396	0.0392	0.1327	0.045*	
C4	0.6329 (2)	0.1148 (3)	0.29612 (19)	0.0412 (5)	
H5	0.6161	0.0243	0.3031	0.049*	
N5	0.9683 (2)	0.6282 (2)	0.31338 (17)	0.0501 (5)	
C1	0.6126 (2)	0.2601 (2)	0.18731 (17)	0.0333 (5)	
C23	1.3156 (2)	1.1510 (3)	1.34542 (19)	0.0412 (5)	
H17	1.3479	1.2442	1.3993	0.049*	
C17	1.1113 (2)	1.0260 (2)	0.8381 (2)	0.0413 (5)	
H14	1.1510	1.1130	0.8989	0.050*	
C9	0.4794 (3)	0.3512 (2)	−0.22784 (19)	0.0421 (5)	
H8	0.3927	0.3077	−0.2846	0.050*	
C15	1.0219 (3)	0.8970 (3)	0.6450 (2)	0.0453 (6)	
H12	1.0026	0.8990	0.5749	0.054*	
C10	0.5971 (3)	0.4366 (2)	−0.24093 (18)	0.0404 (5)	
C24	1.2834 (2)	1.1322 (2)	1.23596 (18)	0.0365 (5)	
H18	1.2933	1.2132	1.2159	0.044*	
C22	1.2991 (3)	1.0296 (3)	1.37339 (19)	0.0453 (6)	
C14	1.0215 (2)	0.7664 (2)	0.75782 (17)	0.0361 (5)	
H11	1.0012	0.6767	0.7662	0.043*	
C19	1.2365 (2)	0.9934 (2)	1.15612 (18)	0.0359 (5)	
O5	0.9102 (3)	0.7052 (2)	0.3672 (2)	0.0828 (7)	
C11	0.7258 (3)	0.5049 (3)	−0.1567 (2)	0.0482 (6)	
H9	0.8045	0.5640	−0.1658	0.058*	
C20	1.2222 (3)	0.8724 (3)	1.1858 (2)	0.0440 (6)	
H15	1.1914	0.7791	1.1323	0.053*	
C18	1.1008 (2)	0.8748 (3)	0.95936 (19)	0.0411 (5)	
C12	0.7364 (3)	0.4844 (2)	−0.05886 (19)	0.0434 (5)	
H10	0.8224	0.5309	−0.0015	0.052*	
O3	1.0717 (3)	0.6017 (3)	0.3604 (2)	0.0869 (7)	
C21	1.2540 (3)	0.8910 (3)	1.2953 (2)	0.0475 (6)	
H16	1.2450	0.8105	1.3159	0.057*	
O4	0.9214 (2)	0.5770 (3)	0.21319 (16)	0.0913 (8)	

### Atomic displacement parameters (Å^2^)

**Table:**

	*U*^11^	*U*^22^	*U*^33^	*U*^12^	*U*^13^	*U*^23^
Ag1	0.05203 (13)	0.04686 (12)	0.03000 (11)	0.00860 (9)	0.00232 (8)	0.00417 (8)
Cl1	0.0777 (5)	0.0579 (4)	0.0394 (3)	0.0099 (3)	0.0166 (3)	0.0252 (3)
Cl2	0.1476 (9)	0.0747 (5)	0.0392 (4)	0.0264 (5)	0.0197 (5)	0.0274 (4)
N2	0.0337 (9)	0.0385 (10)	0.0285 (9)	−0.0010 (8)	0.0017 (8)	0.0120 (8)
O1	0.0446 (9)	0.0506 (10)	0.0358 (9)	−0.0106 (8)	−0.0024 (7)	0.0180 (8)
N3	0.0381 (10)	0.0429 (11)	0.0332 (10)	0.0096 (8)	0.0069 (8)	0.0113 (8)
N1	0.0409 (10)	0.0396 (10)	0.0298 (9)	0.0097 (8)	0.0061 (8)	0.0109 (8)
C6	0.0361 (12)	0.0344 (11)	0.0296 (11)	0.0061 (9)	0.0065 (9)	0.0095 (9)
C7	0.0369 (11)	0.0315 (11)	0.0299 (10)	0.0066 (9)	0.0072 (9)	0.0095 (9)
N4	0.0408 (10)	0.0384 (10)	0.0321 (10)	−0.0039 (8)	0.0060 (8)	0.0115 (8)
C8	0.0357 (12)	0.0432 (13)	0.0367 (12)	0.0035 (10)	0.0078 (10)	0.0163 (10)
C3	0.0421 (13)	0.0488 (14)	0.0359 (12)	0.0122 (11)	0.0085 (10)	0.0207 (11)
C2	0.0355 (11)	0.0345 (11)	0.0335 (11)	0.0086 (9)	0.0077 (9)	0.0130 (9)
C13	0.0273 (10)	0.0388 (12)	0.0352 (11)	0.0065 (9)	0.0086 (9)	0.0109 (10)
O2	0.0791 (14)	0.0669 (13)	0.0411 (10)	−0.0345 (11)	0.0039 (10)	0.0162 (9)
C16	0.0442 (13)	0.0392 (13)	0.0535 (15)	0.0114 (11)	0.0103 (11)	0.0221 (11)
C5	0.0347 (11)	0.0353 (12)	0.0368 (12)	0.0069 (9)	0.0081 (9)	0.0102 (9)
C4	0.0404 (12)	0.0392 (12)	0.0455 (13)	0.0113 (10)	0.0108 (10)	0.0212 (11)
N5	0.0533 (13)	0.0433 (12)	0.0390 (11)	−0.0037 (10)	0.0013 (10)	0.0199 (10)
C1	0.0293 (10)	0.0371 (11)	0.0303 (11)	0.0072 (9)	0.0083 (9)	0.0117 (9)
C23	0.0435 (13)	0.0369 (12)	0.0353 (12)	0.0097 (10)	0.0092 (10)	0.0075 (10)
C17	0.0361 (12)	0.0350 (12)	0.0436 (13)	0.0068 (10)	0.0077 (10)	0.0091 (10)
C9	0.0431 (13)	0.0398 (12)	0.0346 (12)	0.0077 (10)	0.0035 (10)	0.0127 (10)
C15	0.0441 (13)	0.0514 (14)	0.0406 (13)	0.0141 (11)	0.0079 (11)	0.0220 (11)
C10	0.0531 (14)	0.0359 (12)	0.0309 (11)	0.0107 (10)	0.0127 (10)	0.0140 (9)
C24	0.0335 (11)	0.0338 (11)	0.0379 (12)	0.0061 (9)	0.0076 (9)	0.0138 (10)
C22	0.0524 (14)	0.0493 (14)	0.0349 (12)	0.0161 (11)	0.0109 (11)	0.0188 (11)
C14	0.0320 (11)	0.0348 (11)	0.0346 (11)	0.0057 (9)	0.0070 (9)	0.0102 (9)
C19	0.0297 (11)	0.0380 (12)	0.0339 (11)	0.0055 (9)	0.0067 (9)	0.0114 (9)
O5	0.114 (2)	0.0582 (13)	0.0899 (17)	0.0286 (13)	0.0503 (16)	0.0324 (12)
C11	0.0481 (14)	0.0441 (13)	0.0467 (14)	0.0004 (11)	0.0147 (11)	0.0206 (11)
C20	0.0491 (14)	0.0333 (12)	0.0439 (13)	0.0108 (10)	0.0120 (11)	0.0103 (10)
C18	0.0377 (12)	0.0408 (12)	0.0333 (11)	0.0002 (10)	0.0101 (10)	0.0086 (10)
C12	0.0381 (12)	0.0411 (13)	0.0371 (12)	−0.0018 (10)	0.0032 (10)	0.0131 (10)
O3	0.0772 (15)	0.0788 (15)	0.0962 (18)	0.0178 (12)	−0.0082 (13)	0.0520 (14)
C21	0.0557 (15)	0.0424 (13)	0.0474 (14)	0.0157 (11)	0.0136 (12)	0.0227 (11)
O4	0.0622 (13)	0.129 (2)	0.0382 (11)	−0.0217 (13)	−0.0009 (10)	0.0228 (12)

### Geometric parameters (Å, °)

**Table:**

Ag1—N3	2.1467 (19)	C16—C15	1.379 (3)
Ag1—N1	2.1519 (18)	C16—C17	1.379 (3)
Ag1—Ag1^i^	3.2574 (5)	C16—H13	0.9300
Cl1—C10	1.751 (2)	C5—C4	1.377 (3)
Cl2—C22	1.738 (2)	C5—C1	1.386 (3)
N2—C6	1.346 (3)	C5—H6	0.9300
N2—C7	1.419 (3)	C4—H5	0.9300
N2—H1	0.8600	N5—O4	1.224 (3)
O1—C6	1.228 (3)	N5—O3	1.228 (3)
N3—C14	1.339 (3)	N5—O5	1.240 (3)
N3—C15	1.343 (3)	C23—C22	1.377 (3)
N1—C3	1.338 (3)	C23—C24	1.382 (3)
N1—C2	1.348 (3)	C23—H17	0.9300
C6—C1	1.499 (3)	C17—H14	0.9300
C7—C12	1.388 (3)	C9—C10	1.373 (3)
C7—C8	1.389 (3)	C9—H8	0.9300
N4—C18	1.341 (3)	C15—H12	0.9300
N4—C19	1.422 (3)	C10—C11	1.381 (3)
N4—H2	0.8600	C24—C19	1.384 (3)
C8—C9	1.384 (3)	C24—H18	0.9300
C8—H7	0.9300	C22—C21	1.374 (3)
C3—C4	1.381 (3)	C14—H11	0.9300
C3—H4	0.9300	C19—C20	1.387 (3)
C2—C1	1.380 (3)	C11—C12	1.381 (3)
C2—H3	0.9300	C11—H9	0.9300
C13—C14	1.385 (3)	C20—C21	1.383 (3)
C13—C17	1.387 (3)	C20—H15	0.9300
C13—C18	1.499 (3)	C12—H10	0.9300
O2—C18	1.216 (3)	C21—H16	0.9300
			
N3—Ag1—N1	173.41 (7)	C2—C1—C5	118.5 (2)
N3—Ag1—Ag1^i^	100.45 (5)	C2—C1—C6	122.95 (19)
N1—Ag1—Ag1^i^	86.14 (5)	C5—C1—C6	118.50 (19)
C6—N2—C7	127.13 (18)	C22—C23—C24	118.9 (2)
C6—N2—H1	116.4	C22—C23—H17	120.5
C7—N2—H1	116.4	C24—C23—H17	120.5
C14—N3—C15	117.8 (2)	C16—C17—C13	119.1 (2)
C14—N3—Ag1	120.15 (15)	C16—C17—H14	120.5
C15—N3—Ag1	121.49 (16)	C13—C17—H14	120.5
C3—N1—C2	118.20 (19)	C10—C9—C8	119.8 (2)
C3—N1—Ag1	121.23 (15)	C10—C9—H8	120.1
C2—N1—Ag1	119.84 (15)	C8—C9—H8	120.1
O1—C6—N2	124.0 (2)	N3—C15—C16	122.5 (2)
O1—C6—C1	119.57 (19)	N3—C15—H12	118.8
N2—C6—C1	116.38 (18)	C16—C15—H12	118.8
C12—C7—C8	119.4 (2)	C9—C10—C11	120.9 (2)
C12—C7—N2	116.96 (19)	C9—C10—Cl1	119.65 (18)
C8—C7—N2	123.61 (19)	C11—C10—Cl1	119.43 (18)
C18—N4—C19	126.02 (19)	C23—C24—C19	120.3 (2)
C18—N4—H2	117.0	C23—C24—H18	119.9
C19—N4—H2	117.0	C19—C24—H18	119.9
C9—C8—C7	120.0 (2)	C21—C22—C23	121.6 (2)
C9—C8—H7	120.0	C21—C22—Cl2	119.61 (19)
C7—C8—H7	120.0	C23—C22—Cl2	118.78 (19)
N1—C3—C4	122.4 (2)	N3—C14—C13	123.3 (2)
N1—C3—H4	118.8	N3—C14—H11	118.3
C4—C3—H4	118.8	C13—C14—H11	118.3
N1—C2—C1	122.6 (2)	C24—C19—C20	120.0 (2)
N1—C2—H3	118.7	C24—C19—N4	117.7 (2)
C1—C2—H3	118.7	C20—C19—N4	122.3 (2)
C14—C13—C17	118.0 (2)	C12—C11—C10	119.3 (2)
C14—C13—C18	117.1 (2)	C12—C11—H9	120.3
C17—C13—C18	124.7 (2)	C10—C11—H9	120.3
C15—C16—C17	119.3 (2)	C21—C20—C19	119.8 (2)
C15—C16—H13	120.4	C21—C20—H15	120.1
C17—C16—H13	120.4	C19—C20—H15	120.1
C4—C5—C1	119.1 (2)	O2—C18—N4	123.4 (2)
C4—C5—H6	120.5	O2—C18—C13	120.3 (2)
C1—C5—H6	120.5	N4—C18—C13	116.31 (19)
C5—C4—C3	119.2 (2)	C11—C12—C7	120.5 (2)
C5—C4—H5	120.4	C11—C12—H10	119.8
C3—C4—H5	120.4	C7—C12—H10	119.8
O4—N5—O3	119.8 (3)	C22—C21—C20	119.4 (2)
O4—N5—O5	120.0 (3)	C22—C21—H16	120.3
O3—N5—O5	120.1 (3)	C20—C21—H16	120.3
			
Ag1^i^—Ag1—N3—C14	−83.05 (17)	C17—C16—C15—N3	0.5 (4)
Ag1^i^—Ag1—N3—C15	105.37 (18)	C8—C9—C10—C11	−1.5 (4)
Ag1^i^—Ag1—N1—C3	66.84 (17)	C8—C9—C10—Cl1	178.39 (19)
Ag1^i^—Ag1—N1—C2	−103.19 (16)	C22—C23—C24—C19	−0.6 (4)
C7—N2—C6—O1	−3.7 (4)	C24—C23—C22—C21	1.2 (4)
C7—N2—C6—C1	174.4 (2)	C24—C23—C22—Cl2	−176.88 (19)
C6—N2—C7—C12	−164.4 (2)	C15—N3—C14—C13	0.4 (3)
C6—N2—C7—C8	14.4 (4)	Ag1—N3—C14—C13	−171.44 (16)
C12—C7—C8—C9	2.2 (4)	C17—C13—C14—N3	0.2 (3)
N2—C7—C8—C9	−176.6 (2)	C18—C13—C14—N3	176.3 (2)
C2—N1—C3—C4	0.7 (3)	C23—C24—C19—C20	−0.3 (3)
Ag1—N1—C3—C4	−169.49 (18)	C23—C24—C19—N4	−179.3 (2)
C3—N1—C2—C1	−1.3 (3)	C18—N4—C19—C24	−143.7 (2)
Ag1—N1—C2—C1	169.04 (16)	C18—N4—C19—C20	37.3 (4)
C1—C5—C4—C3	−0.5 (3)	C9—C10—C11—C12	1.2 (4)
N1—C3—C4—C5	0.2 (4)	Cl1—C10—C11—C12	−178.6 (2)
N1—C2—C1—C5	1.0 (3)	C24—C19—C20—C21	0.4 (4)
N1—C2—C1—C6	178.6 (2)	N4—C19—C20—C21	179.4 (2)
C4—C5—C1—C2	0.0 (3)	C19—N4—C18—O2	−3.9 (4)
C4—C5—C1—C6	−177.8 (2)	C19—N4—C18—C13	174.3 (2)
O1—C6—C1—C2	−142.3 (2)	C14—C13—C18—O2	−28.4 (3)
N2—C6—C1—C2	39.6 (3)	C17—C13—C18—O2	147.4 (3)
O1—C6—C1—C5	35.4 (3)	C14—C13—C18—N4	153.3 (2)
N2—C6—C1—C5	−142.8 (2)	C17—C13—C18—N4	−30.8 (3)
C15—C16—C17—C13	0.1 (4)	C10—C11—C12—C7	0.8 (4)
C14—C13—C17—C16	−0.4 (3)	C8—C7—C12—C11	−2.5 (4)
C18—C13—C17—C16	−176.2 (2)	N2—C7—C12—C11	176.4 (2)
C7—C8—C9—C10	−0.3 (4)	C23—C22—C21—C20	−1.1 (4)
C14—N3—C15—C16	−0.8 (4)	Cl2—C22—C21—C20	177.0 (2)
Ag1—N3—C15—C16	170.97 (18)	C19—C20—C21—C22	0.2 (4)

Symmetry codes: (i) −*x*+2, −*y*+1, −*z*+1.

### Hydrogen-bond geometry (Å, °)

**Table:**

*D*—H···*A*	*D*—H	H···*A*	*D*···*A*	*D*—H···*A*
N2—H1···O4	0.86	2.10	2.953 (3)	169
N4—H2···O1^ii^	0.86	2.10	2.931 (3)	162
C2—H3···O5	0.93	2.51	3.210 (3)	133
C3—H4···O3^i^	0.93	2.57	3.300 (3)	136
C4—H5···Cl2^iii^	0.93	2.83	3.516 (3)	132
C5—H6···O1^iv^	0.93	2.55	3.376 (3)	148
C8—H7···O1	0.93	2.27	2.841 (3)	119
C11—H9···O2^v^	0.93	2.49	3.194 (4)	132
C16—H13···O5^vi^	0.93	2.48	3.370 (4)	160
C20—H15···O2	0.93	2.46	2.906 (3)	109

Symmetry codes:  (ii) *x*+1, *y*+1, *z*+1; (i) −*x*+2, −*y*+1, −*z*+1; (iii) −*x*+2, −*y*+1, −*z*+2; (iv) −*x*+1, −*y*, −*z*; (v) *x*, *y*, *z*−1; (vi) −*x*+2, −*y*+2, −*z*+1.
